# Family structure and health, how companionship acts as a buffer against ill health

**DOI:** 10.1186/1477-7525-5-61

**Published:** 2007-11-23

**Authors:** Amelia R Turagabeci, Keiko Nakamura, Masashi Kizuki, Takehito Takano

**Affiliations:** 1 International Health Section, Division of Public Health, Graduate School of Tokyo Medical and Dental University, Tokyo 113-8519, Japan; 2 Health Promotion Section, Division of Public Health, Graduate School of Tokyo Medical and Dental University, Tokyo 113-8519, Japan

## Abstract

**Background:**

Health and well-being are the result of synergistic interactions among a variety of determinants. Family structure and composition are social determinants that may also affect health behaviours and outcomes. This study was performed to examine the associations between family structure and health and to determine the protective effects of support mechanisms to improve quality of health outcome.

**Methods:**

Six hundred people, selected by multistage sampling to obtain a representative population of men and women aged 20–60 living in communities in Japan, were included in this study. Data regarding subjective views of one's own health, family structure, lifestyle and social support were collected through structured face-to-face interviews on home visits. Systolic and diastolic blood pressures, height and weight were measured by trained examiners. The associations between family structure and health after controlling for demographics, lifestyle and social support were examined using logistic and linear regression analyses.

**Results:**

Subjects living alone were significantly more likely to be in ill health, as determined using the General Health Questionnaire, in comparison to those in extended families (OR = 3.14). Subjects living alone or as couples were significantly more likely to suffer from severe hypertension in comparison to those living in extended families (OR = 8.25, OR = 4.90). These associations remained after controlling for the influence of lifestyle. Subjects living only with spouse or in nuclear family had higher probabilities of mental ill health in the absence than in the presence of people showing concern for their well-being.

**Conclusion:**

The results of this study infers that a support mechanism consisting of companionship and the presence of family or other people concerned for one's well being acts as a buffer against deleterious influence of living in small family that will lead to improved quality of health outcome.

## Background

Over the last several decades, marked changes have occurred in the family structure in many societies by various factors, such as migration, economic fluctuations, and instability. The World Health Organisation (WHO) defines health determinant as "the range of personal, social, economic and environmental factors which determine the health status of individuals or population" [1]. Therefore, family composition is regarded as a health determinant in our social environment. Health and well-being of an individual are mainly the results of synergistic interactions among a number of determining factors.

Family structure often refers to the diversity of types of family unit composition. Although the term 'family' often refers to the conventional family unit consisting of biological parents and children, commonly known as nuclear family, others include single-parent family or even couple-only family. Other family types include living alone, couple with no children and extended family structure including cohabiting relatives.

The distribution of such family structures vary among countries with the proportions of couple-only families and single parent families with dependent children increasing, while proportion of couple families with children have decreased, particularly those with dependent children [2]. Between 1991 and 2000, there was a decrease in number of nuclear family units with an increase in single parent family units in the USA, and a similar trend was observed in Australia between 1976 and 2001 [3,4]. For the period between 1979 and 1998, there was a decline in number of couples with dependent children from 31 to 23% and an increase from 4 to 7% in single parent families in the UK [5]. In Japan, there was an increase in single-person households from 1990 to 2005 [6]. The trend in family structures in developing countries are also varying, with extended family structures being replaced by nuclear families resulting in increases in number of elderly people living alone [7].

Increases in single and couple-only family structures have been observed worldwide, not only in developed but also in developing countries [2,7]. In 1991, 51.1% of North American family households had no one under 18 living with them. The 1993 Basic Survey on National Life by the Ministry of Health and Welfare reported that childless families accounted for 65 percent of all families in Japan [8]. The increase in 'alone' and 'couple only' family types is synonymous with the increase in aging population and decline fertility rate experienced in many countries with increases in delaying marriage.

There are concerns how changes in family structures influence the health and well-being and quality of health outcome of the population. While some studies examined parent-child relationship and health [9-13], there have been few studies of multigenerational composition effect. Previous studies have indicated association of large family composition and health behaviours in different communities [14,15]. There are concerns regarding the further influences of multigenerational family composition on measures of health status and quality of life.

Some studies have also suggested that there are associations between multigenerational family composition and supportive environments [16-18]. It would be of interest to explore the possible impacts of supportive environments prevalent in multigenerational families on quality of life health measures.

The present study was performed to examine the association between family structure and health measures and to determine how these associations vary according to differences in lifestyles and social support networks to support healthy quality of life outcomes.

## Methods

### Study Sample

A cross-sectional survey among 600 people in Japan selected by a multistage sampling to obtain a representative population of men and women aged 20–60 living in communities. All participants gave their informed consent to participation in the study, which was performed with the approval of the local authorities. Detailed information on site selection and participation criteria have been reported elsewhere [19,20].

Face-to-face interview were conducted by 36-trained interviewers through home visits using a structured questionnaire. Of the 387 subjects who completed the interview with valid data, the response rates were 49.2% men and 58.2% women. For the purpose of this study, analysis focused on 386 subjects consisting of 183 men and 203 women.

### Measurements

#### Health measures

Health-related quality of life measures included perceived physical fatigue, mental stress, and health concern. The Japanese version of the 12 items General Health Questionnaire (GHQ 12) [21] was also used for further assessment of mental health status, with a score ≥ 4 taken to indicate poor mental status.

Blood pressure, height and weight were measured to obtain physiological parameters. Systolic and diastolic blood pressure (sbp and dbp, respectively) were measured using portable standardized electronic blood pressure gauges (#HEM629; Omron, Tokyo, Japan). The measurements were categorised into mild to moderate hypertension as [sbp 140~179 mmHg or dbp 90~109 mmHg] and severe hypertension as [sbp ≥ 180 mmHg and dbp ≥ 110 mmHg] according to the Healthy Japan 21 standards developed by Ministry of Health, Labour, and Welfare [22]. Body mass index [BMI] was determined as height divided by weight squared [BMI = height (m)/weight^2 ^(kg^2^)]. BMI, systolic and diastolic blood pressure were entered as continuous variables.

#### Family structure characteristics

Family structure are generally categorised into nuclear families [married with/without children] and non-nuclear families or multiple families, as described previously [14,17]. Other classifications are comprised of married couples with or without children and married couples with/without children living with their parents or multiple family units [18,23]. To identify particular differences in influences on health measures in comparison with multigenerational families, the following definition of family structures were used: *Alone *– Single person household; *Couple *– with no children, or siblings sharing the same domicile; *Nuclear family *– conventional family of parent(s) and child(ren); and *Extended family *– family of grandparent(s), parent(s) and child(ren) [3 or more generation].

For the purpose of this study, 'alone' and 'couple' family will be referred to as 'small' families.

#### Lifestyle and social support

Having regular meals is defined as taking each of three meals – breakfast, lunch, and supper – almost everyday. The Healthy Japan 21 [22] recommends alcohol consumption per day as 1 *ghou *sake (1 unit) equivalent to approximately 633 ml of beer and 1 standard glass of wine, while 500 ml of beer is equivalent to 0.8 units. In this study, the frequency of drinking alcohol is given in days per week, and subjects who drank alcohol more than 2 days per week were defined as regular alcohol drinker. Other lifestyle variables included current smoking, regular exercise and healthy eating habits – i.e., having a well-balanced diet, moderate salt intake and avoiding overeating.

Social support are defined as various form of supports by family members, friends, colleagues and other people in social networks individuals are belong to. In this study, we evaluated companionship at meals determined using the question 'With who you have meals?', the existence of concerned person (family or others) when faced with a stressful situation and having supper at home by 8 pm; the latter was included as a measure of how much time is spent with family.

### Statistical analysis

Statistical analyses were performed using SPSS 14.0 [SPSS Inc; Chicago, IL, USA]. Descriptive analyses were performed to determine the distribution of population by absolute number and percentage. In logistic regression, categorical health measures were entered as dependent variables while systolic and diastolic blood pressure and BMI as continuous dependent variables in linear regression. In the first model, only demographic variables were adjusted when testing the association between family structure and health. Subsequent models were adjusted for age, sex and lifestyle. Interaction between family structure and social support was examined by stratification according to the presence or absence of social support using different variables.

## Results

### Health status by family structures

The demographic, health related quality of life and physiological health measures were shown in Table 1 indicated that smaller family structures included a larger percentage of people aged 50 and above (66.7%), more men than women and approximately 76% were classified as belonging to the 'alone' family structure. The proportion of perceived subjective health status (perceived physical fatigue, mental stress and health concern) 'very often' was also high in the alone family structure, with high levels of poor mental health (34.5%) and severe hypertension (20.5%). Nuclear family showed a higher proportion of non-hypertensive subjects (54%).

**Table 1:** Age, sex and health status of subjects by family structure

		**^#^Family Structure**							
		
		**Alone (n = 29)**		**Couple (n = 58)**		**Nuclear family (n = 190)**		**Extended family (n = 109)**	
		
		No.	%	No.	%	No.	%	No.	%
**Demographics**									
Age	20	6	20.7	1	1.7	34	17.9	12	11.0
	30	4	13.8	6	10.3	50	26.3	20	18.3
	40	5	17.2	7	12.1	45	23.7	27	24.8
	50	7	24.1	19	32.8	39	20.5	28	25.7
	60	7	24.1	25	43.1	22	11.6	22	20.2
									
Sex	Male	22	75.9	33	56.9	72	37.9	56	51.4
	Female	7	24.1	25	43.1	118	62.1	53	48.6
									
**Health status**									
Perceived Physical Fatigue	Very often	4	13.8	3	5.2	24	12.6	6	5.5
	Sometimes	13	44.8	26	44.8	86	45.3	57	52.3
	Seldom	11	37.9	23	39.7	67	35.3	36	33.0
	None	1	3.4	6	10.3	13	6.8	10	9.2
									
Perceived Mental Stress	Very often	7	24.1	6	10.3	32	16.8	13	11.9
	Sometimes	14	48.3	22	37.9	93	48.9	56	51.4
	Seldom	6	20.7	20	34.5	33	17.4	25	22.9
	None	2	6.9	10	17.2	32	16.8	15	13.8
									
Perceived Health Concern	Very often	3	10.3	6	10.3	9	4.7	7	6.4
	Sometimes	12	41.4	27	46.6	89	46.8	57	52.3
	Seldom	13	44.8	22	37.9	70	36.8	27	24.8
	None	1	3.4	3	5.2	22	11.6	18	16.5
									
Mental Health Status (measured by GHQ)	Poor [GHQ 4]	10	34.5	7	12.1	39	20.6	18	16.8
	Good [GHQ < 4]	19	65.5	51	87.9	150	79.4	89	83.2
									
*Hypertension	Severe [sbp 180 and dbp 110]	6	30.0	12	29.3	8	6.2	4	5.5
	Others	14	70.0	29	70.7	121	93.8	69	94.5
									
	Hypertensive [sbp 140 or dbp 90]	11	55.0	31	75.6	60	46.5	40	54.8
	Others*	9	45.0	10	24.4	69	53.5	33	45.2
									
		**Mean**	**S.D**	**Mean**	**S.D**	**Mean**	**S.D**	**Mean**	**S.D**
	Systolic blood pressure	148.00	26.98	158.59	28.90	136.40	22.72	138.21	22.43
	Diastolic blood pressure	95.85	17.66	94.34	16.11	85.99	13.64	88.32	11.37
Body Mass Index		21.30	4.54	23.08	2.99	22.23	3.04	22.11	2.79

^#^Family Structure: composition in one household unit

Alone – single-person household;

Couple – with no children or siblings sharing the same domicile

Nuclear family – conventional family of parent(s) and child(ren).

Extended family – family consisting of grandparent(s), parent(s), and child(ren) [3 or more generations]

*Hypertension – included only 263 subjects for whom systolic and diastolic blood pressures were measured.

Mild/moderate hypertension (sbp 140–179 or dbp 90–109), Severe Hypertension (sbp 180 and dbp 110), Others* – (sbp < 140 and dbp < 90)

The association between family structure and health-related quality of life or physiological measurements are shown in Table 2. Family structures showed significant associations with measures of hypertension and poor mental health as measured by GHQ. Compared to extended families, subjects living in small families showed increase likelihood of severe hypertension and poor mental health, with OR: 8.25 (living alone) and 4.90 (couple) p < 0.01, and 3.14 (living alone) [95% CI: 1.21–8.15] p < 0.02, respectively. Significant association were also observed between blood pressure coefficients of beta; systolic and diastolic blood pressure was higher by 12.5 mm/Hg (p < 0.01) and by 7.11 mm/Hg (p < 0.05) in couple and alone family structures, respectively, as compared with those in extended families.

**Table 2:** Age and sex adjusted for associations between health status and family structure

	**Alone**			**Couple**			**Nuclear family**			**Extended family**
		
	**OR**	**95% CI**	***p *value**	**OR**	**95% CI**	***p *value**	**OR**	**95% CI**	***p *value**	
**Health-related Quality of Life Measures**										
Perceived Physical Fatigue										**Ref.**
Very often	2.78	0.72 – 10.75	0.139	1.02	0.24 – 4.33	0.979	2.36	0.92 – 6.03	0.074	**1.0**
Perceived Mental Stress										
Very often	2.50	0.88 – 7.11	0.086	0.95	0.33 – 2.70	0.920	1.38	0.68 – 2.79	0.372	
Perceived Health Concern										
Very often	1.58	0.37 – 6.66	0.533	1.38	0.42 – 4.46	0.594	0.84	0.30 – 2.35	0.733	
Mental Health Status										
Poor [GHQ 4]	3.15	1.20 – 8.15	0.018	0.82	0.31 – 2.14	0.678	1.11	0.59 – 2.09	0.740	
**Physiological Health Measures**										
Hypertension										
Severe Hypertension [sbp 180 and dbp 110]	8.25	1.87 – 36.35	0.005	4.90	1.41 – 17.09	0.013	1.61	0.45 – 5.79	0.466	
Hypertensive [sbp 140 or dbp 90]	0.85	0.27 – 2.64	0.778	1.62	0.63 – 4.13	0.313	1.21	0.62 – 2.35	0.579	
										
	**β**	**[95% CI] mm/Hg**	***p *value**	**β**	**[95% CI] mm/Hg**	***p *value**	**β**	**[95% CI] mm/Hg**	***p *value**	
		
Systolic blood pressure	9.86	-0.28 – 20.00	0.057	12.54	4.65 – 20.42	0.002	4.30	1.70 – 10.3	0.159	**Ref. 0.0**
Diastolic blood pressure	7.11	0.57 – 13.66	0.030	3.30	-1.88 – 8.39	0.200	0.14	-3.74 – 4.02	0.940	
Body Mass Index	-1.04	-2.28 – 0.21	0.102	0.48	-0.51 – 1.47	0.342	0.47	-0.26 – 1.19	0.205	

### Health status by family structure, adjusted by lifestyle

Table 3 shows daily lifestyle practices and behaviours according to family structure. Couple families show the highest proportion of having regular meals daily, healthy eating habits and regular exercise. Extended family structures show healthier lifestyle practices than 'alone' family structures, which include the highest proportions of regular alcohol drinkers and current smokers and the lowest proportions of subjects with regular exercise (27.6%).

**Table 3:** Lifestyle of the subjects by family structure

		**Alone (n = 29)**		**Couple (n = 58)**		**Nuclear family (n = 190)**		**Extended family (n = 109)**	
					
		No.	%	No.	%	No.	%	No.	%
Regular meals	Breakfast	18	62.1	52	89.7	151	79.5	96	88.1
	Lunch	25	86.2	57	98.3	184	96.8	108	99.1
	Supper	27	93.1	58	100	186	98.4	102	93.6
Smoking Status	Current Smoker	17	58.6	19	32.8	62	32.6	38	34.9
Regular drinker	> 2 days/week	18	62.1	25	43.1	66	34.7	45	41.3
Regular exercise		8	27.6	29	50	83	43.7	44	40.4
Healthy eating habits		12	41.4	44	75.9	123	64.7	70	64.2

Regular drinker – those who drink alcohol more than 2 days a week

As shown in Table 4, the associations between family structure and health measures persists even after controlling for effects of lifestyle practices. The likelihood of poor mental health was more than double in the 'alone' family structures and that of severe hypertension was greater among small family structures taking into account current lifestyle practices as compared to extended families. The family structure effect was stronger in the risks of severe hypertension among small families as compared to the reference group [nuclear and extended family structures]. Current smoking status showed significantly higher risks in 'alone' and in 'couple' family structure. Similar trends were observed for the risk of regular alcohol consumption.

**Table 4:** Associations between health status and family structure adjusted by age, sex, and lifestyles

	**Family structure**									
	
**Health Status**	**Alone**			**Couple**			**Nuclear family**			**Extended family**
		
Lifestyle variable used for adjustment	**OR**	**95% CI**	***p *value**	**OR**	**95% CI**	***p *value**	**OR**	**95% CI**	***p *value**	
										**Refrence 1.0**
**Mental Health Status – Poor**										
Regular meals, breakfast	2.37	0.09 – 6.37	0.088	0.76	0.28 – 2.03	0.586	0.97	0.50 – 1.87	0.930	
Regular meals, lunch	2.66	1.00 – 7.04	0.049	0.80	0.30 – 2.12	0.650	1.07	0.56 – 2.03	0.830	
Regular meals, supper	3.31	1.18 – 8.13	0.019	0.80	0.30 – 2.11	0.653	1.10	0.58 – 2.08	0.770	
Current Smoker	3.09	1.19 – 8.01	0.021	0.82	0.31 – 2.15	0.683	1.11	0.58 – 2.08	0.758	
Regular alcohol drinker	3.03	1.16 – 7.91	0.023	0.82	0.31 – 2.16	0.688	1.10	0.58 – 2.08	0.759	
Regular exercise	3.17	1.22 – 8.22	0.018	0.81	0.31 – 2.14	0.670	1.11	0.59 – 2.08	0.760	
Healthy eating habits	2.97	1.14 – 7.73	0.026	0.84	0.32 – 2.20	0.720	1.12	0.59 – 2.10	0.734	
										
**Severe hypertension **[sbp >= 180, dbp >= 110]										
Regular meals, breakfast	8.06	1.83 – 35.42	0.006	4.87	1.39 – 17.02	0.013	1.61	0.45 – 5.84	0.465	
Regular meals, lunch	11.15	2.43 – 51.11	0.002	4.93	1.41 – 17.28	0.013	1.65	0.46 – 5.97	0.443	
Regular meals, supper	8.17	1.85 – 36.04	0.006	4.87	1.40 – 16.98	0.013	1.61	0.45 – 5.78	0.467	
Current Smoker	11.55	2.43 – 54.97	0.002	5.30	1.49 – 18.84	0.010	1.85	0.50 – 6.77	0.355	
Regular alcohol drinker	7.66	1.69 – 34.63	0.008	5.26	1.48 – 18.65	0.010	1.62	1.45 – 5.89	0.463	
Regular exercise	7.93	1.76 – 35.75	0.007	4.89	1.38 – 17.03	0.013	1.60	0.45 – 5.75	0.471	
Healthy eating habits	7.78	1.75 – 34.62	0.007	5.00	1.43 – 17.51	0.012	1.62	0.45 – 5.84	0.462	

Individual ORs were calculated by using a health status (poor mental health status or severe hypertention) as a dependent variable and three variables (age, sex, and one of the lifestyle variable) as inidependent variables.

The systolic blood pressure among couple families was 12 mm/Hg higher than that in extended families. The association with diastolic blood pressure was weaker although it was also significant in the alone family structure after controlling for lifestyle variables with the exception of healthy eating behaviour.

### Health status by family structure, stratified by social support measures

Table 5 shows the existence of social support mechanism among different family structures. The proportion of subjects having meals with companionship was higher among 'couple', 'nuclear' and 'extended' family units particularly for breakfast and supper. The same was also true for supper at home by 8 pm Pacific Time. The alone family structure had highest percentage of subjects reporting the absence of people concerned about their welfare when confronted with a stressful situation (48%) and these subjects were likely to eat supper late (55%).

**Table 5:** Characteristics of familial social support by family structure

		**Alone (n = 29)**		**Couple (n = 58)**		**Nuclear family (n = 190)**		**Extended family (n = 109)**	
		
		No.	%	No.	%	No.	%	No.	%
Breakfast companionship	Alone	20	69.0	11	19.0	44	23.2	15	13.8
	Family	1	3.4	40	69.0	123	64.7	88	80.7
	Others	0	0.0	2	3.4	0	0.0	0	0.0
									
Lunch companionship	Alone	10	34.5	22	38.6	56	29.9	19	17.4
	Family	0	0.0	21	36.8	48	25.7	40	36.7
	Others	17	58.6	14	24.6	83	44.4	50	45.9
									
Supper companionship	Alone	23	79.31	7	12.1	18	9.5	9	8.3
	Family	1	3.45	51	87.9	170	89.5	100	91.7
	Others	1	3.45	0	0	2	1.1	0	0
									
Supper at home	by 8 pm	11	37.93	43	74.1	144	75.79	92	84.40
	after 8 pm	16	55.17	15	25.9	46	24.2	17	15.6
									
Existence of caring person	Yes	15	51.7	48	82.8	160	84.2	94	86.2
	No	14	48.3	10	17.2	30	15.8	15	13.8

Table 6 shows association between health status and family structure stratified by social support. Similar associations between family structure and health measures were observed when the subjects were stratified by meal companionship. Interaction was observed for existence of caring person and family structure in relation to the association to mental health status.

**Table 6:** Associations between health stautus and family structure adjusted by age, sex, and familial support

	**Family structure**									
	
**Health Status**	**Alone**			**Couple**			**Nuclear family**			**Extended family**
		
Groups defined by familial support variables	**OR**	**95% CI**	***p *value**	**OR**	**95% CI**	***p *value**	**OR**	**95% CI**	***p *value**	
**Mental Health Status – Poor**										
Breakfast companionship										**Reference 1.0**
some companionship	-		-	0.67	0.20 – 2.25	0.516	0.76	0.35 – 1.66	0.496	
none	3.44	0.57 – 20.78	0.178	1.70	0.19 – 14.87	0.630	1.74	0.33 – 9.23	0.510	
Lunch companionship										
some companionship	2.53	0.73 – 8.75	0.140	0.98	0.28 – 3.37	0.977	1.12	0.54 – 2.33	0.770	
none	2.73	0.49 – 15.21	0.252	0.56	0.11 – 2.95	0.490	0.75	0.20 – 2.77	0.083	
Supper companionship										
some companionship	-	-	-	0.84	0.29 – 2.37	0.740	1.16	0.59 – 2.25	0.671	
none	1.65	0.27 – 10.05	0.589	0.53	0.04 – 7.65	0.643	0.70	0.09 – 4.95	0.791	
Supper time										
at home by 8 pm	5.04	1.31 – 19.40	0.019	0.84	0.28 – 2.58	0.762	1.12	0.55 – 2.25	0.757	
other	2.14	0.41 – 11.24	0.370	0.67	0.09 – 4.77	0.687	0.98	0.23 – 4.23	0.980	
Existence of caring person										
Yes	5.55	1.71 – 17.96	0.004	0.47	0.14 – 1.52	0.206	0.94	0.48 – 1.83	0.847	
No	3.09	0.24 – 39.74	0.387	8.11	0.69 – 95.65	0.096	4.26	0.46 – 39.31	0.201	
										
**Severe hypertension**										
Breakfast companionship										**Reference 1.0**
some companionship	-		-	3.60	1.22 – 10.67	0.020				
none	14.93	1.50 – 148.40	0.020	6.35	0.48 – 83.10	0.160				
Lunch companionship										
some companionship	10.65	2.15 – 52.78	0.004	3.33	1.05 – 10.61	0.040				
none	10.60	0.74 – 152.40	0.080	10.34	1.04 – 103.27	0.050				
Supper time										
at home by 8 pm	2.77	0.46 – 16.52	0.264	4.15	1.52 – 11.30	0.005				
other	-			-						
Existence of caring person										
Yes	8.31	1.52 – 45.44	0.020	3.77	1.39 – 10.30	0.009				
No	11.46	0.95 – 138.70	0.550	2.90	0.15 – 56.05	0.470				

Individual ORs were calculated by using a health status (poor mental health status or severe hypertention) as a dependent variable and two variables (age and sex) as inidependent variables. ORs were calculated each group defined by individual familial support variables.

"-" stands for ORs were not calculated because of small number of subjects in that category.

## Discussion

This study was performed to determine the effect of family structure on health-related quality of life and physiological health measures and to observe how these associations vary according to differences in lifestyles and familial social support mechanism for quality of health outcome. The effects of family structure on measures of health-related quality of life and hypertension persist regardless of age, gender, lifestyle risk factors and social support. The existence of concerned/caring persons buffers deleterious influence of living in small family compared to multigenerational families and lead to improved mental health status.

A strength of this study was the use of representative of the study population, although the sample size is relatively small. Another strength was that the present study assimilated activities with perceived health conditions and support mechanisms. Inclusion of measures of family quality time suggested the importance of such opportunities to improve health status. Further strength of the study was the use of actual measurements of systolic and diastolic blood pressure, and assessment of varying degrees of hypertension status associated with different family structures. The study design adopted here shared certain limitations with other cross-sectional study, and caution should be used when making causal inferences on outcome in relation to associated daily life patterns. The cross-sectional study design precludes direct assessment of intra-individual changes and restricts inferences to group averages, results being contemporaneous.

Studies demonstrated a wide range of ways in which extended family communication is associated with the adoption of various patterns of eating [15,16]. These studies indicated a need for opportunities to learn together and communicate ways to improve nutrition behaviour, with the extension of participants beyond parent and child relationship to promote healthy eating habits. It has also been confirmed that daily life activities also show relations to health [24,25].

A number of studies have also indicated associations between health and both the support system and family social network [14,26-29] although they did not discuss time spent with family in relation to positive health outcome. Eating supper at home regularly by 8 pm was introduced as a measure of social support, to assess quality time spent with family, which has a positive influence on health-related behaviour.

A study in Japan [18] showed that multigenerational family settings provides a number of rewards, including social support, prestige, greater control and power within the family for women, and another study confirmed that the availability of helpers in the household eases domestic burdens [23]. In a study of the differences between self-perceived health and functional health status among men and women in Canada [30], living in nuclear family unit was shown to be associated with better health among men than women, than other family structures. These observations indicate the importance of family structure as a predictor of health.

Although living in large family structures is viable, it is often precluded by various situations as family members move in search of opportunities, marriage or migration. The association between large family structures and better health outcomes was in contrast to the findings of other studies. A study of survival of Afro-American ESRD patients and the role of family structure indicated that females living in a complex family structure have double the mortality risk than those living in a simple family structure and were overburdened by demands of family and disease [31]. Another study indicated no association between smoking and family structure [32] while men in 'sandwich' families (extended families in the present study) had a higher likelihood of smoking [23]. According to the current results, when a caring person exists, alone family structure was strongly associated with poor mental health status; while none of caring persons exist, people in couple families are more likely to become poor mental health status. This suggests a lack of caring person even nominally living with family members strongly deteriorates quality of life and existence of caring person buffers associations between small family structure and quality of health outcome.

There are some studies to discuss smaller family structures that include 'alone' and 'couple' family showed higher probabilities of poor health status measures as compared to extended families. Such associations in small family structures could be linked to current lifestyle practices, while in the extended family structure, the existence of network of support within the family composition plays a role in the reduced measures of poor health status.

The complex composition of large family structures will enhance awareness and more health consciousness, with chances for exchange of health information. In this study, extended families were those comprised of three or more generations, and therefore transfer of information will be prevalent as a common tradition amongst these family units. Many daily activities and lifestyle are closely related to people living together in larger family groups, and therefore large family structures are more likely to facilitate a traditional Japanese lifestyle, which is regarded as healthier. In this sense, such a family structure composition acts as a buffers against ill health inferring support for healthy quality of life outcomes.

## Conclusion

Living in multi-generational family structures should be viewed not only as a social obligation but also as a useful form of social support to better health-related quality of life. The result of the present study demonstrated the inference that a large family structure can act as a buffer against certain deleterious health outcome of daily life activities and lifestyle, and that it is an effective medium for the transfer of health-related information.

## List of abbreviations

BMI: Body mass index;

CI: Confidence interval;

dbp: Diastolic blood pressure;

GHQ: General Health Questionnaire;

OR: Odds ratio;

sbp: Systolic blood pressure;

WHO: World Health Organization.

## Competing interests

The author(s) declare that they have no competing interests.

## Authors' contributions

ART conceived the study, design and data analysis and drafted the manuscript. KN participated in the study design and helped in the draft manuscript. MK participated in the statistical analysis and interpretation. TT participated in the design of the study and coordination. All authors read and approved the final manuscript.
